# Discovery of non-covalent rhinovirus 3Cpro inhibitors by molecular docking, *in vitro* assays, molecular dynamics simulations and DFT analyses

**DOI:** 10.3389/fphar.2025.1560571

**Published:** 2025-05-16

**Authors:** Susu Zhang, Keli Zong, Jiajun Ruan, Xiaojing Liu, Xu Zhao, Youzhi Zhang, Chun Hu, Xingzhou Li

**Affiliations:** ^1^ Key Laboratory of Structure-Based Drug Design & Discovery (Ministry of Education), Shenyang Pharmaceutical University, Shenyang, China; ^2^ Beijing Institute of Pharmacology and Toxicology, Beijing, China; ^3^ Department of Hepatology, Fifth Medical Center of Chinese PLA General Hospital, Beijing, China

**Keywords:** human rhinovirus, 3Cpro, molecular docking, molecular dynamics, DFT

## Abstract

Human rhinovirus 14 (HRV-14) is a leading cause of the common cold, with its 3C protease (3Cpro) playing a crucial role in viral replication by cleaving polyproteins into functional proteins and enzymes. This makes 3Cpro a promising target for therapeutic intervention. In this study, to identify novel HRV-14 3Cpro non-covalent inhibitors, we performed a virtual screening of the TopScience and TargetMol (United States) database and selected 44 potential compounds for HRV-14 3Cpro inhibitory activity evaluation. Preliminary assays at 50 μM showed that compounds **S21**, **S33**, **S34**, and **S43** exhibited inhibition rates of 80.51%, 96.5%, 75.59%, and 88.79%, respectively. Further characterization revealed that **S21** and **S34** exhibited moderate activity with IC_50_ values of 30.40 ± 0.67 μM and 24.11 ± 0.55 μM, respectively, while **S33** and **S43** displayed stronger inhibition with IC_50_ values of 11.32 ± 0.71 μM and 2.33 ± 0.5 μM, respectively. To elucidate the binding mode of **S33** and **S43** to HRV-14 3Cpro, we conducted all-atom molecular dynamics (MD) simulations and density functional theory (DFT) calculations on the docked complexes of compounds **S33** and **S43** with HRV-14 3Cpro. MD analyses, including principal component analysis (PCA), free energy landscapes (FEL), and dynamic cross-correlation matrices (DCCM), revealed that both compounds enhanced the structural stability of the HRV-14 3Cpro while reducing its flexibility and internal dynamics. These findings suggested that **S33** and **S43** are promising candidates for optimization and clinical development as novel non-covalent HRV-14 3Cpro inhibitors.

## Graphical Abstract

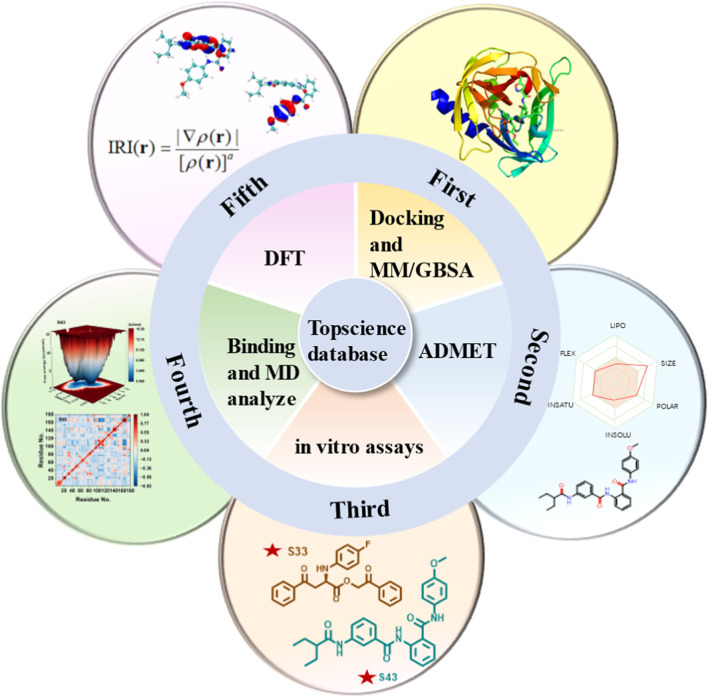

## 1 Introduction

Human rhinovirus 14 (HRV-14) is a leading pathogen responsible for acute respiratory infections, including common colds and asthma exacerbations, as well as chronic conditions such as bronchiolitis and pneumonia (Jacobs et al., 2013; Gern, 2010). HRV-14 is a 30-nm positive-sense single-stranded RNA virus that belongs to the picornavirus family. Upon infection, the viral RNA genome translates into a single polyprotein, which is processed by the viral proteases 2A and 3C cysteine protease (Palmenberg et al., 2009; Tapparel et al., 2007; Esneau et al., 2019). This processing generates essential capsid and replicative proteins. The 3C cysteine protease (3Cpro) enzyme, characterized by its chymotrypsin-like fold and shallow active site, has a strong preference for the cleavage sequence Gln/Gly Pro. Its highly conserved active site and substrate specificity facilitate key protein hydrolysis events during the HRV-14 replication cycle, making 3Cpro a crucial target for antiviral treatment (Matthews et al., 1994; Fan et al., 2020; Jensen et al., 2015).

**FIGURE 1:**
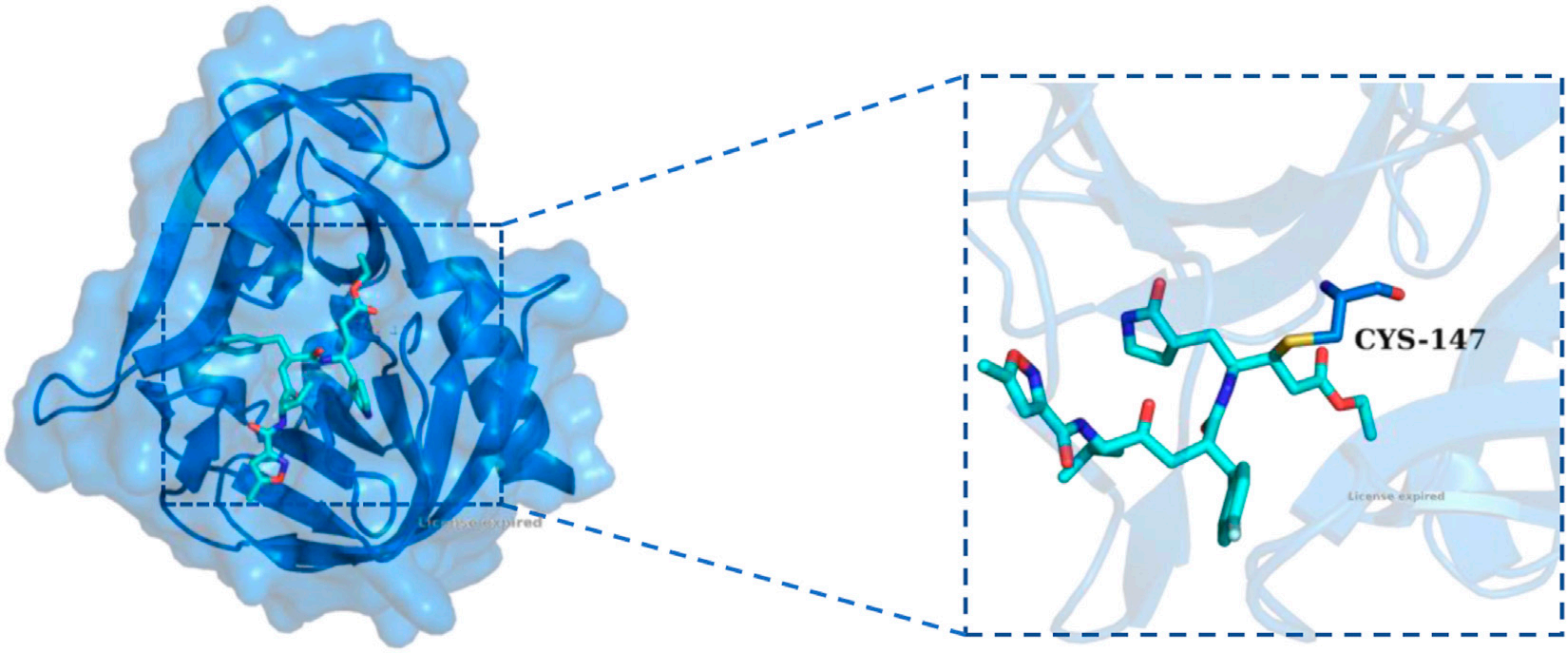
The X-ray structure of Rupintrivir covalently bound to HRV-14 3C protein (PDB ID: 1CQQ). The overall structure of HRV-14 3Cpro is shown as a blue cartoon with a transparent surface, and Rupintrivir was displayed in stick representation (cyan). The inset on the right highlights the covalent interaction between Rupintrivir and the catalytic residue CYS-147, demonstrating the irreversible covalent bonding mechanism.

**FIGURE 2:**
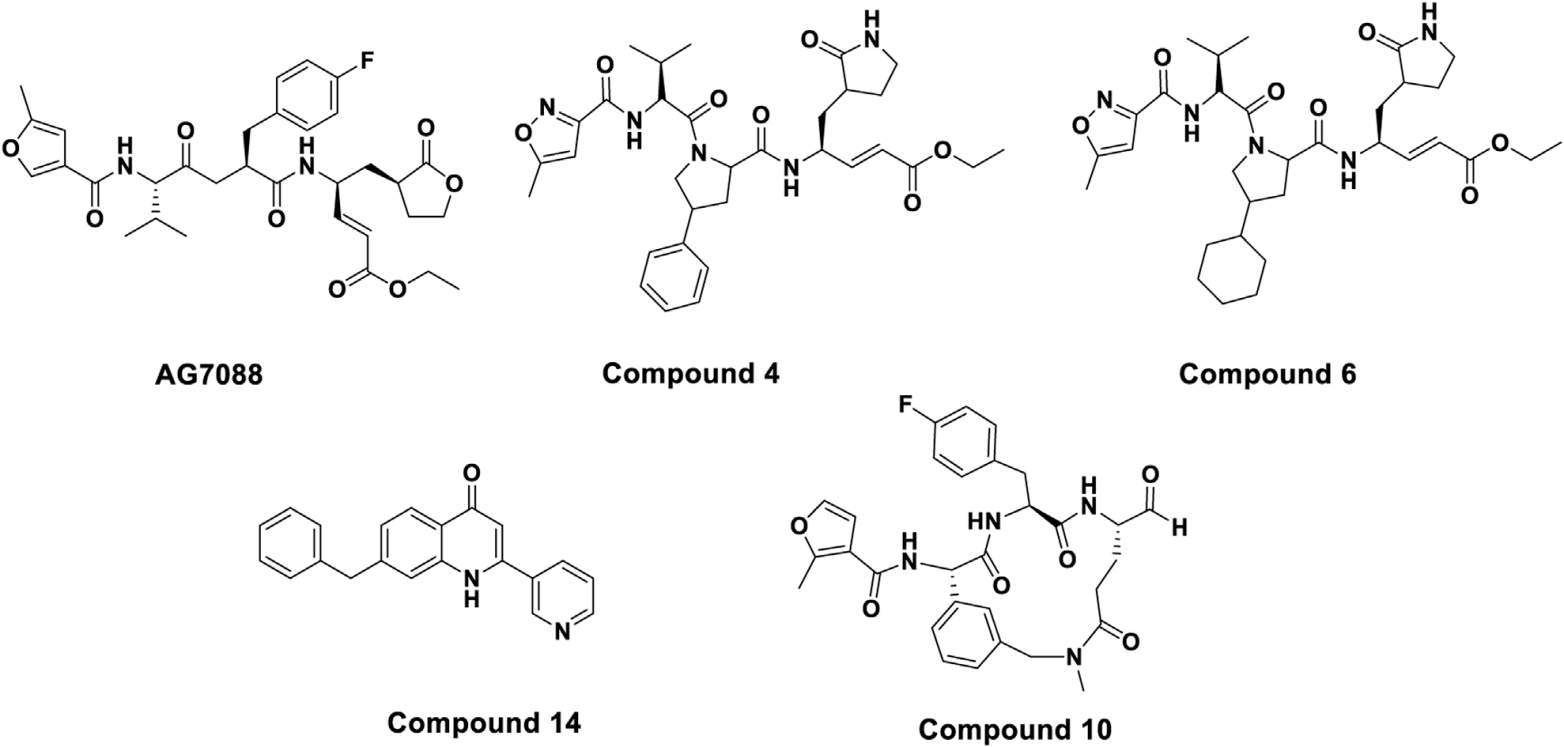
Chemical structures of some known HRV-14 3Cpro inhibitors.

Although the majority of previously reported HRV-14 3C protease inhibitors are covalent inhibitors, which form irreversible bonds with the protease active site (Figure 1), our study specifically focused on identifying non-covalent inhibitors. Among them, Rupintrivir (AG7088) has received a lot of attention for its excellent antiviral activity (EC_50_ = 12 nM). However, due to its poor pharmacokinetic performance, it could not be further advanced in clinical studies (Binford et al., 2007; Zalman et al., 2000). Although derivatives based on the Rupintrivir structure (**compounds 4 and 6**) showed similar antiviral activity, macrocyclic inhibitors with optimized P1 and P3 residues (compound 10, IC_50_ = 0.008 µM) showed lower sub micromolar activity. However, as 3Cpro covalent inhibitors, they pose potential risks due to their irreversible binding mechanisms, which can lead to off-target effects and long-term toxicity. In contrast, non-covalent inhibitors bind reversibly, offering the potential for greater selectivity, improved safety, and tunable pharmacokinetics. Despite these advantages, few non-covalent inhibitors of HRV-14 3Cpro have been reported, and those that exist are limited in structural diversity and potency (Kawatkar et al., 2016; Namoto et al., 2018). Baxter et al. identified for the first time a class of non-covalent inhibitors through fragment-based screening that demonstrated effective blocking of viral replication while reducing off-target effects (**compound 14**, pIC_50_ = 4.7) (Baxter et al., 2011). This research advance provides a safer and more effective treatment option for HRV-14 infections and suggests that non-covalent inhibitors hold promise as important candidates for future drug development. The structures of the above compounds are shown in Figure 2.

**FIGURE 3:**
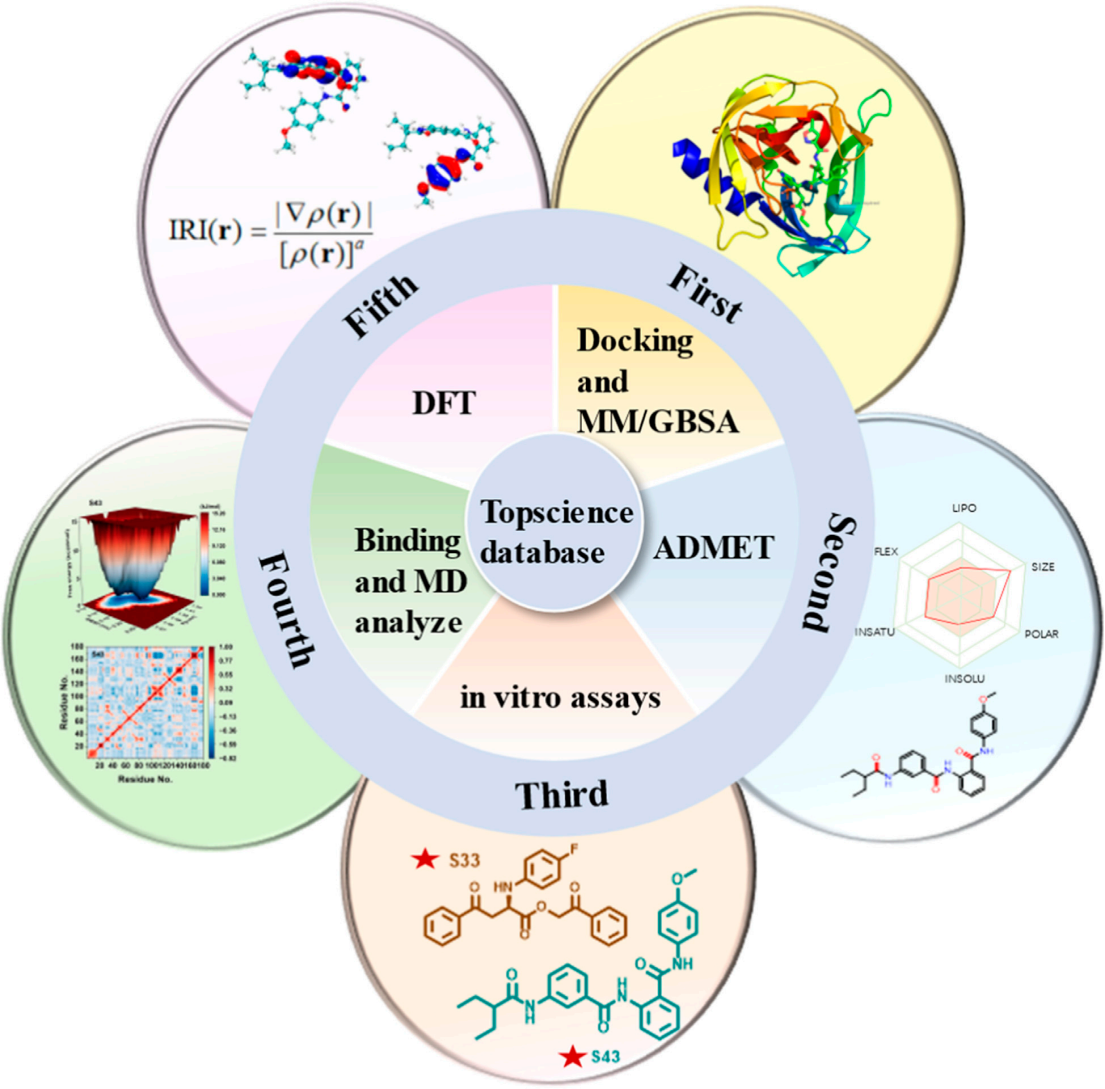
Multi-step computational and experimental workflow for identifying HRV-14 3Cpro inhibitors. The process includes: molecular docking and MM/GBSA analysis, ADMET prediction, *in vitro* enzymatic assays, molecular dynamics simulations, and DFT and IRI calculations.

Due to the lack of commercially available therapeutic drugs for HRV-14 and the associated risks of off-target effects and toxicity with 3Cpro covalent inhibitors, this study aimed to promote the development of anti-HRV-14 drugs. Starting from 3Cpro non-covalent inhibitors, we discovered new structures of 3Cpro non-covalent inhibitors against HRV-14 from existing compound libraries. The study was initially based on the composite crystal structure of 3Cpro and compound 6 (PDB: 5fx6) and employed a non-covalent docking method to virtually screen 10 million compounds from the Topscience database, ultimately narrowing down to 5 million candidate compounds. Following further docking studies and MM/GBSA calculations, these compounds were refined to 44 types and subjected to ADMET predictions to evaluate their overall safety. Subsequently, through in-depth *in vitro* evaluations, four candidate compounds were identified, with particular emphasis on compounds **S33** and **S43**. Finally, to investigate the binding modes and structural stability of 3Cpro with compounds **S33** and **S43**, we conducted full atomic molecular dynamics (MD) simulations and density functional theory (DFT) calculations. Through this comprehensive research strategy (Figure 3), we successfully identified the structures of two non-covalent HRV-14 3Cpro inhibitors, thereby laying a scientific foundation for the development of antiviral drugs targeting HRV-14.

## 2 Materials and methods

### 2.1 Protein preparation and grid generation

The crystal structure of HRV-14 3Cpro complexed with compound6 (PDB ID: 5FX6) with a resolution of 1.45Å was retrieved from the Protein Database PDB (https://www.rcsb.org). This structure was prepared using the Glide protein preparation guide in Schrödinger 2023-4 version, which involves the following key steps: firstly, missing hydrogen atoms were added to ensure the integrity of the structure; Secondly, unnecessary water molecules and other impurities are removed from the structure; Subsequently, the orientation of the side chains was optimized to improve the accuracy of binding with ligands; Finally, the energy of the structure was minimized by using the OPLS4 force field to ensure that its geometric configuration matches its intrinsic conformation under physiological conditions. In addition, based on the optimized protein, a three-dimensional grid was generated around the active site, and its size and shape were adjusted according to the characteristics of the active site. These preparation steps significantly increase the likelihood of successfully screening effective drug candidate molecules (Kar, 2025).

### 2.2 Preparation of ligand library

To conduct virtual screening, compounds were initially selected from the TopScience and TargetMol (United States) database as starting candidates. The TopScience and TargetMol (United States) database was selected due to its diverse and well-curated library of drug-like small molecules, many of which are commercially available and have favorable physicochemical properties suitable for structure-based virtual screening. Additionally, this database has been widely used in early-stage hit discovery studies, making it a practical and reliable source for identifying potential lead compounds. A preliminary screening was performed based on Lipinski’s rules, which facilitated the elimination of compounds with potential adverse pharmacokinetic properties. Following this initial selection, detailed structural processing of the screened compounds was carried out using the Create Phase Database module in Schrödinger 2023-4, alongside the OPLS_2005 force field. To account for ligand flexibility, multiple low-energy conformers were generated for each compound using LigPrep, and these conformations were used as input for Glide XP docking. This processing involved several critical steps, including energy minimization, generation of three-dimensional structures, evaluation of ionization states, and tautomer generation, thereby ensuring that each compound’s structure was both accurate and biologically relevant. Ultimately, this approach yielded a high-quality ligand library, providing a robust foundation for the subsequent virtual screening experiments.

### 2.3 Compounds library screening

In the conducted study, an optimized library of proteins and ligands was utilized for a systematic screening process using the Virtual Screening Workflow module in Schrödinger 2023-4. High-throughput virtual screening (HTVS) was employed to swiftly identify the top 10% of compounds with the most favorable scores. These selected compounds underwent standard precision (SP) docking to enhance their potential interactions with the target proteins. Subsequently, the top 10% of compounds from SP docking served as starting points for ultra precision (XP) docking, allowing for a more accurate prediction of the optimal binding modes. The binding affinity was evaluated using the molecular mechanics/generalized Born surface area (MM/GBSA) method within the Prime module, where free binding energy was calculated as a measure of the strength of protein-ligand interactions. To ensure accurate energy minimization, the VSGB solvation model and the OPLS4 force field were applied, stabilizing molecular structures in a physiologically relevant environment. Additionally, strain energy calculations were performed to assess the adaptability of the ligands to the protein’s binding site; lower strain energy values indicated enhanced binding stability. This comprehensive screening process ensured that the selected compounds exhibited strong protein-ligand interactions, favorable binding affinities, and structural adaptability, thereby providing a robust foundation for identifying potential therapeutic candidates.

### 2.4 ADMET properties

The ADMET properties of all hits were analyzed by using SwissADME (http://www.swissadme.ch/) and ProTox 3.0 (https://tox.charite.de/protox3/) web facilities. SwissADME is used to predict the pharmacokinetic properties of compounds, including absorption, distribution, metabolism, and excretion. ProTox 3.0 is used to evaluate the toxicity characteristics of candidate compounds, which can predict oral toxicity, hepatotoxicity, cytotoxicity, and potential adverse reactions (Daina et al., 2017; Jamrozik et al., 2024).

### 2.5 Inhibitory activity from *in vitro* assays

In the *in vitro* cleavage assay, the peptide Dabcyl-EALFQGPPKFE-Edans was identified as the most efficiently processed substrate by HRV-14 3Cpro (Acro, Beijing, China), and was subsequently utilized in the enzyme inhibition assay. A total of 44 candidate compounds were purchased from Topscience (TargetMol, United States). Dimethyl sulfoxide (DMSO) (Innochem, Beijing, China) served as the solvent for dissolving the compounds, and all solutions were stored at −20°C. The assays were conducted using 100 μL reaction samples, which comprised 1 μL of protease, 10 μL of a 10 μM substrate solution, and varying concentrations of the tested compounds (ranging from 50 μM to 0.617 μM) in a 50 mM Tris buffer at pH 7.0. To evaluate the inhibitory effects of the compounds, the protease and each compound were preincubated at 37°C for 30 min before the addition of the substrate. The reaction mixture was subsequently incubated for 1 h at 37°C. Fluorescence measurements were performed using a multifunction enzyme label reader (SpectraMax M5, Molecular Devices), set to an excitation wavelength of 340 nm and an emission wavelength of 490 nm. Inhibition rates were determined at single points, and half maximal inhibitory concentration (IC_50_) values were calculated using Prism software (n = 5). The inhibition rate was quantified according to the following formula:

$$Inhibition\;rate\;\left(100\%\right)=\frac{{\text{RFU}}_{\text{emzyme}}-{\text{RFU}}_{\text{sample}}}{{\text{RFU}}_{\text{emzyme}}-{\text{RFU}}_{\text{control}}}\times 100\%$$

### 2.6 Molecular dynamics simulations

The GROMACS 2020.7 beta suite was utilized for MD simulations to analyse the dynamic binding behaviour and stability of protein-candidate compound complexes. The simulations were conducted over a duration of 200 ns at a temperature of 298.15 K, employing the Amber ff14SB force field. Ligand parameters were generated using the General Amber Force Field (GAFF), with the fitted charges calculated at the B3LYP/cc-pVDZ level using Gaussian 16, Multiwfn, and Sobtop software. For each system, solvation was achieved in a cubic box with a 10 Å edge length, filled with TIP3P water molecules. The genion module in GROMACS was employed to introduce the necessary counterions (Na^+^ and Cl^−^) to neutralize the system. Energy minimization was performed under periodic boundary conditions utilizing the steepest descent algorithm for 5,000 steps. Electrostatic interactions were computed using the Particle Mesh Ewald (PME) method. To control the temperature of the system, the V-rescale temperature coupling approach was applied under the NVT ensemble, progressively raising the temperature from 0 K to 298.15 K. Subsequently, the Parrinello-Rahman barostat was employed in the NPT ensemble to maintain the system pressure at 1 atm. Finally, each system underwent a 200 ns molecular dynamics simulation to ensure that equilibrium and stability were achieved (Wang et al., 2004; Frisch et al., 2016; Lu and Chen, 2012; Lu, 2022).

### 2.7 DFT calculations

DFT calculations were conducted to assess the electronic density and energy characteristics of the molecules. The computational analysis was performed using the Gaussian 16 software package, while GaussView 6.0 was utilized for visualization purposes. Molecular geometries were optimized at the M06-2X/def2-TZVP level of theory without imposing any symmetry constraints. To better simulate the biological environment, all calculations were conducted using the SMD implicit solvation model with water as the solvent. The M06-2X functional was selected for DFT calculations due to its high accuracy in describing non-covalent interactions such as dispersion, hydrogen bonding, and π–π stacking, which are critical for protein–ligand binding studies. Compared to more traditional functionals like B3LYP, M06-2X offers better performance for thermochemistry and kinetics in systems involving main-group elements. It also outperforms ωB97XD in some cases when applied to non-covalent complexes with mixed interaction types. The def2-TZVP basis set was chosen to provide a good balance between computational efficiency and accuracy in describing electron distribution and molecular orbitals. This level of theory has been widely used in studies involving biological ligands and receptor interactions. The compounds were modeled as having a neutral charge and a singlet multiplicity. Electrostatic potential maps and the energy gaps between the Highest Occupied Molecular Orbital (HOMO) and Lowest Unoccupied Molecular Orbital (LUMO) were derived from the optimized structures using Multiwfn software (Zhao and Truhlar, 2009; Silvarajoo et al., 2020).

## 3 Results and discussion

### 3.1 Molecular docking

In Molecular docking, as a powerful computational tool, was crucial for identifying potential candidate drugs and played a key role in developing effective therapeutic methods. Prior to large-scale screening, docking protocol validation was carried out by re-docking the co-crystallized ligand into the active site of HRV-14-14 3Cpro. The RMSD between the crystallographic pose and the docked pose was 0.5230 Å, indicating that the docking protocol accurately reproduced the experimental binding conformation (Supplementary Figure S1). This study employed high-precision protein for non-covalent docking. Firstly, the compounds were preliminarily screened using XP GScore to predict their binding affinity with the target protein. Subsequently, MM/GBSA binding free energy calculations were performed on the compounds with the best XP GScore to provide more accurate estimates of binding affinity. In addition, further evaluation of strain energy was conducted to identify more stable and rational molecular conformations, as lower strain energy was often considered advantageous.

Through the above steps, compounds with XP GScore values better than −5.0 kcal/mol were selected for further analysis. This threshold was chosen based on the docking score of the reference non-covalent inhibitor, **Compound 14**, which yielded an XP GScore of −5.226 kcal/mol under identical docking conditions. By selecting compounds with comparable or better scores, we ensured that only candidates with predicted binding affinities equal to or stronger than the reference compound were retained. Meanwhile, the binding free energy evaluated by the MM/GBSA method showed that all 44 top candidate compounds had values below −15 kcal/mol, indicating their strong binding ability. Except for **S27** with an MM/GBSA value of 17.37 kcal/mol, which was lower than the reference compound, all other compounds demonstrated significant binding stability, indicating that these candidate compounds were expected to exhibit good stability. Strain energy analysis further revealed that the reference compound had the lowest binding energy (0.04 kcal/mol), which was superior to the other 44 top candidate compounds. In this analysis, compounds **S10** and **S8** had the smallest binding energies to overcome, at 0.58 kcal/mol and 0.91 kcal/mol, respectively. The values of the remaining compounds were also below 13.01 kcal/mol. This discovery suggested that these compounds may have undergone minimal conformational changes during binding, thereby maintaining a more favorable binding state. Overall, these findings highlighted the potential advantages of candidate compounds in terms of energetics and binding ability, making them powerful candidates for further development and optimization. The binding advantages demonstrated by these compounds laid a solid foundation for subsequent experimental validation and emphasized their potential applications in the development of anti-HRV-14 drugs (Supplementary Table S1).

### 3.2 ADMET analysis

**FIGURE 4:**
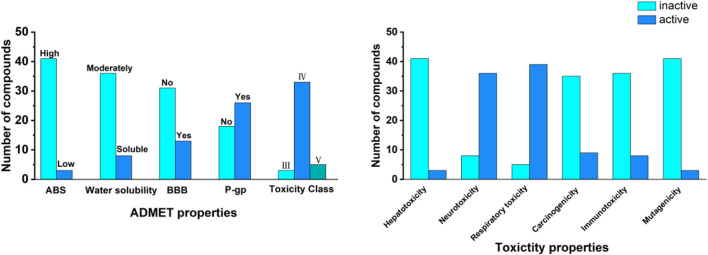
ADMET properties and toxicity profiles of the 44 top compounds. The left panel summaries pharmacokinetic properties, including gastrointestinal absorption (ABS), water solubility, blood–brain barrier (BBB) permeability, P-glycoprotein (P-gp) substrate status, and toxicity class. The right panel displays predicted toxicity profiles, including hepatotoxicity, neurotoxicity, respiratory toxicity, carcinogenicity, immunotoxicity, and mutagenicity. Data were classified as “active” or “inactive” based on computational predictions.

In the field of drug discovery, a comprehensive assessment of the ADMET properties of compounds was crucial for evaluating their potential as candidate drugs. Therefore, this study utilized the web-based software SwissADME and ProTox 3.0 to conduct pharmacokinetic and safety analyses of the selected candidate compounds. By analyzing the predicted ADMET and toxicity characteristics of candidate compounds, as shown in Figure 4, the results indicate that most compounds exhibit high absorption and moderate solubility and can be considered as candidate compounds. In terms of toxicity classification, a significant portion of the compounds belonged to Categories III and IV, corresponding to moderate and low toxicity, respectively, while compounds classified under Category V were relatively few. Regarding toxicological characteristics, the analyzed compounds were primarily assessed as inactive concerning hepatotoxicity and neurotoxicity. In contrast, it was found that most compounds exhibited respiratory toxicity and carcinogenicity. Immunotoxicity and mutagenicity demonstrated the lowest levels of activity, suggesting a reduced risk in these categories. These findings highlighted the potential of these compounds as 3Cpro inhibitors while also identifying potential safety concerns. Overall, this assessment provided valuable insights for the early screening of promising candidate drugs (Supplementary Tables S2, S3).

### 3.3 *In vitro* assays analysis

**FIGURE 5:**
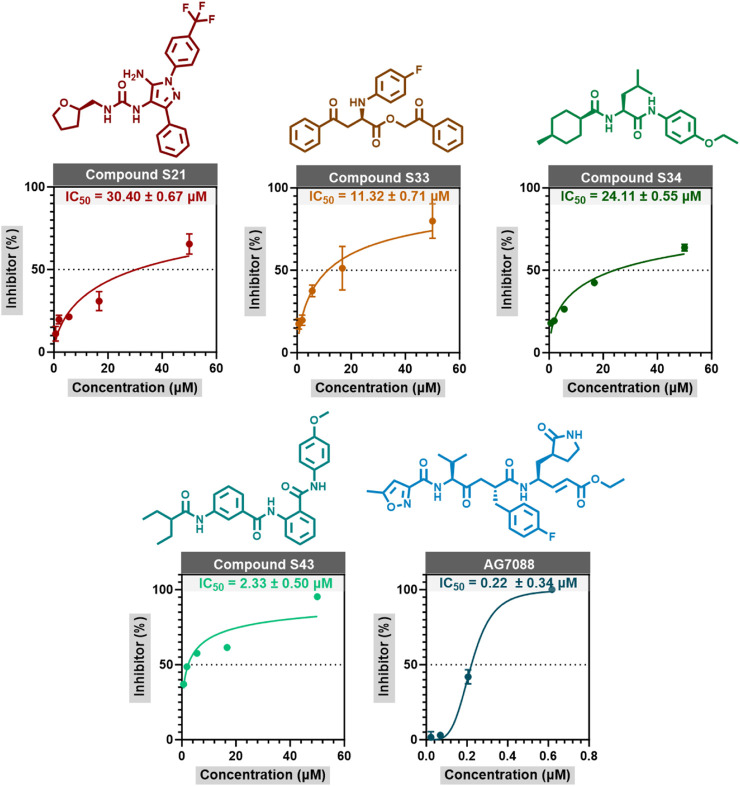
The concentration response curves of S21, S33, S34, and Rupintrivir are measured through enzyme inhibition experiments. The data in the figure is represented as mean ± standard error (mean ± SEM).

An *in vitro* activity assay was conducted on 44 advanced candidate compounds, with the inhibitor concentration set at 50 μM during the initial cleavage experiments. The results showed that the inhibition rates of compounds **S21**, **S33**, **S34**, and **S43** were 80.51%, 96.5%, 75.59%, and 88.79%, respectively, all exceeding 70%. These findings indicated that these compounds were promising anti-HRV-14 candidates for further research. The IC_50_ values of these compounds were determined through concentration-response experiments, and the relevant concentration-response curves and data were shown in Figure 5. **S33** and **S43** exhibited excellent activity, with IC_50_ values of 11.32 ± 0.71 μM and 2.33 ± 0.5 μM, respectively, which were much lower than the IC_50_ values of the other candidate compounds, **S21** and **S34** (30.40 ± 0.67 μM and 24.11 ± 0.55 μM, respectively). Although the IC_50_ value of the reference drug **Rupintrivir** was 0.22 ± 0.34 μM, indicating higher inhibitory activity, **S33** and **S43**, as novel non-covalent inhibitors, had different mechanisms of action in their design. This difference made them important directions for candidate drug development. With further optimization, they were expected to provide new treatment options for HRV-14. This discovery provided a new opportunity and theoretical basis for the development of antiviral drugs (Supplementary Table S4).

### 3.4 Molecular dynamics simulation

MD simulations were conducted to further investigate the influence of explicit solvent molecules on the protein, with a particular focus on fluctuations and conformational adjustments. Additionally, the simulations provided time-averaged properties of the complex system across different timescales, offering insights into molecular interactions. Based on a comprehensive evaluation of computational and pharmacological data, compounds **S33** and **S43** were identified as promising candidates. To assess their stability and dynamic behavior in complex with HRV-14 3Cpro, MD simulations were performed on both the apo-3Cpro (ligand-free 3Cpro) and the 3Cpro-ligand complexes of **S33** and **S43**. These simulations aimed to gain a deeper understanding of the potential of **S33** and **S43** as effective HRV-14 inhibitors.

#### 3.4.1 Structural deviations and compactness

**FIGURE 6:**
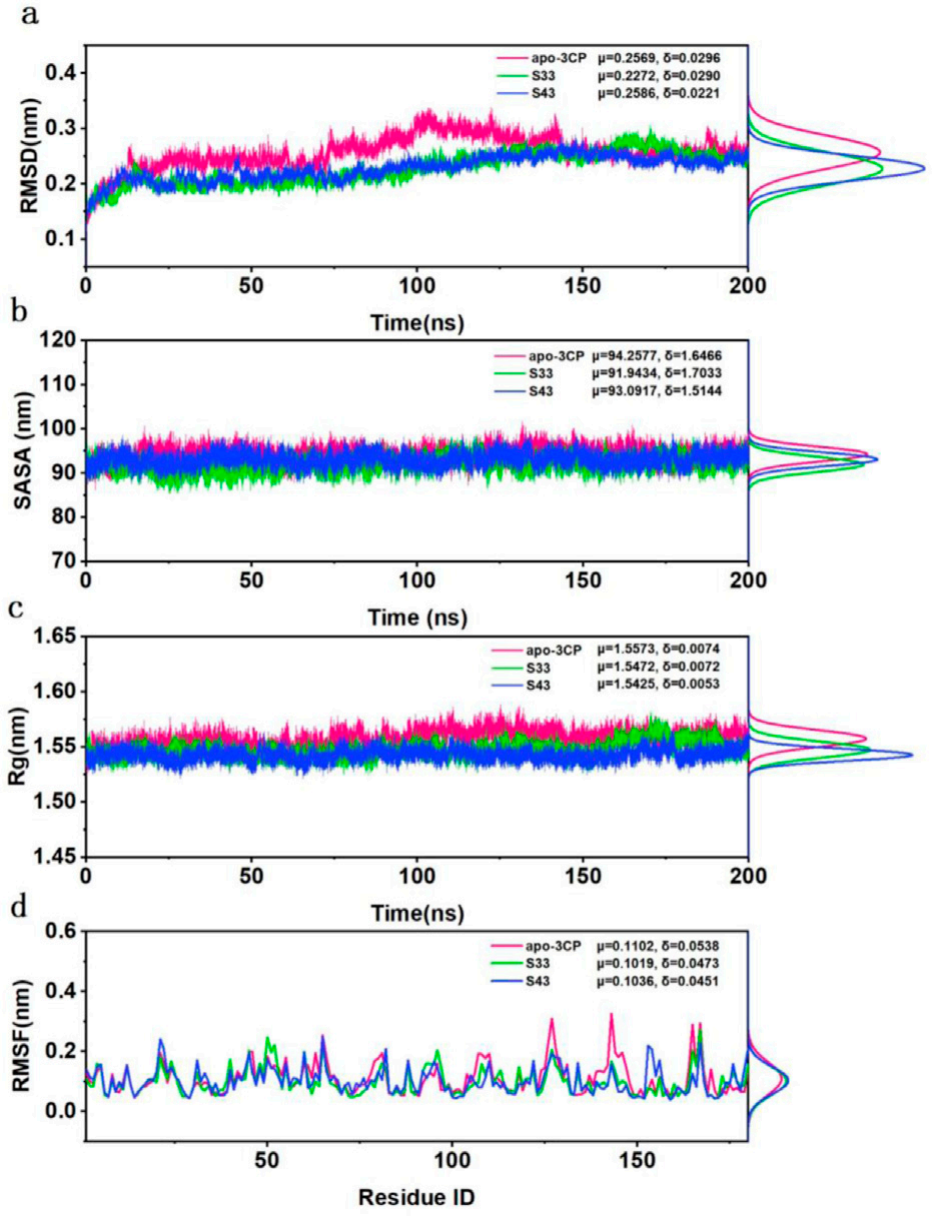
Structural stability and flexibility analysis of HRV-14 3Cpro in the apo form (pink) and in complex with **S33** (green) and **S43** (blue) over 200 ns of MD simulation. **(a)** The RMSD for apo-3Cpro, **S33** and **S43** along with their respective distributions. **(b)** The SASA for apo-3Cpro, **S33** and **S43** along with their respective distributions. **(c)** The SASA for apo-3Cpro, **S33** and **S43** along with their respective distributions. **(d)** The RMSF for apo-3Cpro, **S33** and **S43** along with their respective distributions.

The analysis of root means square deviation (RMSD), solvent accessible surface area (SASA), radius of gyration (Rg), and root mean square fluctuation (RMSF) provided insights into the structural stability and dynamic characteristics of the studied systems. The RMSD curves (Figure 6a) indicated that all systems reached equilibrium within approximately 50 ns and maintained stability throughout the 200 ns simulation. The mean RMSD values for apo-3Cpro, **S33**, and **S43** were 0.2569 nm, 0.2272 nm, and 0.2586 nm, respectively, with standard deviations of 0.0296 nm, 0.0290 nm, and 0.0221 nm, respectively. The slightly higher mean RMSD of apo-3Cpro compared to the **S33** and **S43** complexes suggested greater conformational fluctuations in the absence of ligand binding.

The SASA values (Figure 6b) exhibited a relatively consistent trend across the systems, with the apo-3Cpro system showing slightly higher surface exposure than the ligand-bound states. The mean SASA values for apo-3Cpro, **S33**, and **S43** were 94.2577 nm^2^, 91.9434 nm^2^, and 93.0917 nm^2^, respectively, with standard deviations of 1.6466 nm^2^, 1.7033 nm^2^, and 1.5144 nm^2^. This observation implied that the protein structure became more compact upon binding of these compounds. Similarly, the Rg curves (Figure 6c) demonstrated consistent compactness across all systems throughout the simulation, with minimal differences in mean values. The apo-3Cpro system exhibited slightly larger Rg values (μ = 1.5573 nm, δ = 0.0074 nm), whereas the mean Rg values for **S33** and **S43** were 1.5472 nm and 1.5425 nm, respectively, with standard deviations of 0.0072 nm and 0.0053 nm. This further confirmed that the Rg values of the apo-3Cpro system were higher than those of the ligand-bound states, indicating a more extended conformation in the absence of ligands.

Finally, the RMSF data (Figure 6d) revealed that fluctuations were primarily concentrated near specific residue regions, with the apo-3Cpro system displaying higher fluctuations in some flexible loop regions. The mean RMSF values for apo-3Cpro, **S33**, and **S43** were 0.1102 nm, 0.1019 nm, and 0.1036 nm, respectively, with standard deviations of 0.0538 nm, 0.0473 nm, and 0.0451 nm. The increased horizontal mobility of residues in the apo state suggested that ligand binding contributed to structural stability and reduced local flexibility. Overall, the comparative analysis indicated that ligand binding resulted in more stable and compact protein structures, as evidenced by lower RMSD, SASA, and Rg values, and reduced flexibility at the residue level. These findings suggested that compounds **S33** and **S43** could act as effective inhibitors, providing a solid foundation for further experimental validation and therapeutic development.

#### 3.4.2 PCA analysis

**FIGURE 7:**
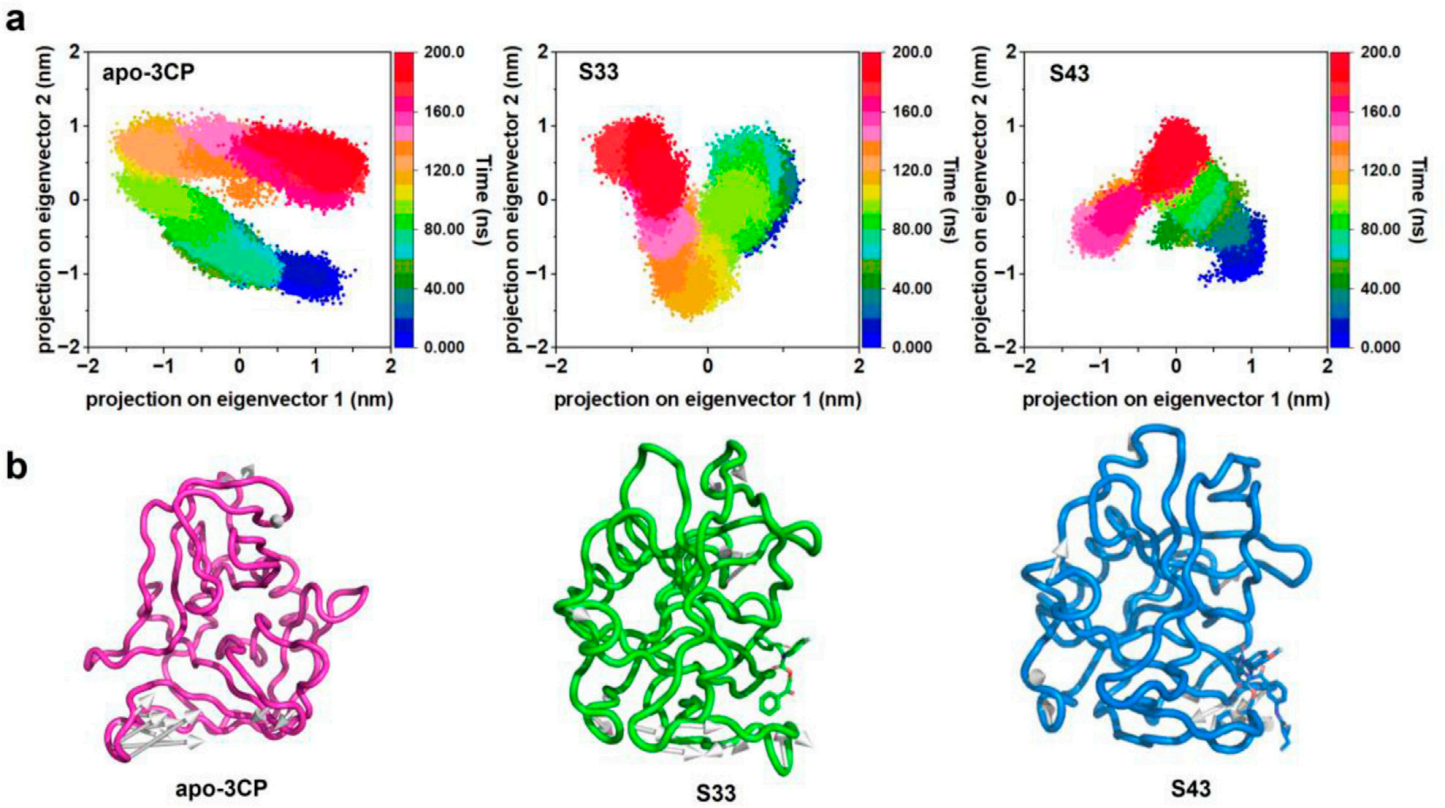
PCA of HRV-14 3Cpro in apo form and in complex with **S33** and **S43**. **(a)** Representation of the 2D projections of apo-3Cpro, **S33** and **S43** complexes conformational changes during the simulation. **(b)** Projection of the first principal component onto the protein structure to visualize the motion direction of each residue.

Principal component analysis (PCA) was utilized to analyze the four-dimensional projection of the conformational space sampled from apo-3Cpro, **S33**, and **S43**, as depicted in Figure 7a. This analysis aimed to gain a deeper understanding of the overall conformational changes and stability of receptor-ligand complexes during the simulation process. The projection of the first two eigenvectors revealed distinct conformational spaces for each complex. The apo-3Cpro structure demonstrated a wider and more flexible conformation, consistent with previous observations that, in the absence of ligand binding, the protein remains in a relatively dynamic and exploratory state, potentially sampling multiple conformations. Such widespread movement, particularly in flexible regions, could lead to reduced binding efficiency and structural instability. Compared to apo-3Cpro, the **S33** complex showed a more compact and well-defined structure, indicating that **S33** effectively stabilizes the protein and diminishes conformational flexibility. This reduction in dynamics, especially in functionally critical regions, may enhance binding efficiency and overall inhibitory potential, underscoring the role of ligands in promoting structural rigidity. **S43** exhibited even more pronounced compactness than apo-3Cpro and **S33**, suggesting excellent stability and the potential to lock the protein into favorable binding conformations. The observed tight packing indicated strong interactions between the ligand and the protein, further enhancing its inhibitory effect. The comparison of these three structures highlighted the significant impact of ligand binding on protein stability and kinetics. Both **S33** and **S43** significantly imparted substantial stability to the protein structure compared to apo-3Cpro.

The projection of the first principal component onto the protein structure (Figure 7b) offered a visualization of the dominant motion within the system. Apo-3Cpro exhibited a broader range of movements, especially in certain flexible areas. However, these movements were significantly reduced in the presence of compounds **S33** and **S43**, indicating that these compounds conferred substantial stability to the protein structure. This stabilizing effect is particularly important in regions critical to protein function, where reduced flexibility may enhance the binding efficiency and overall inhibitory potential of the compounds.

#### 3.4.3 FEL and DCCM analysis

**FIGURE 8:**
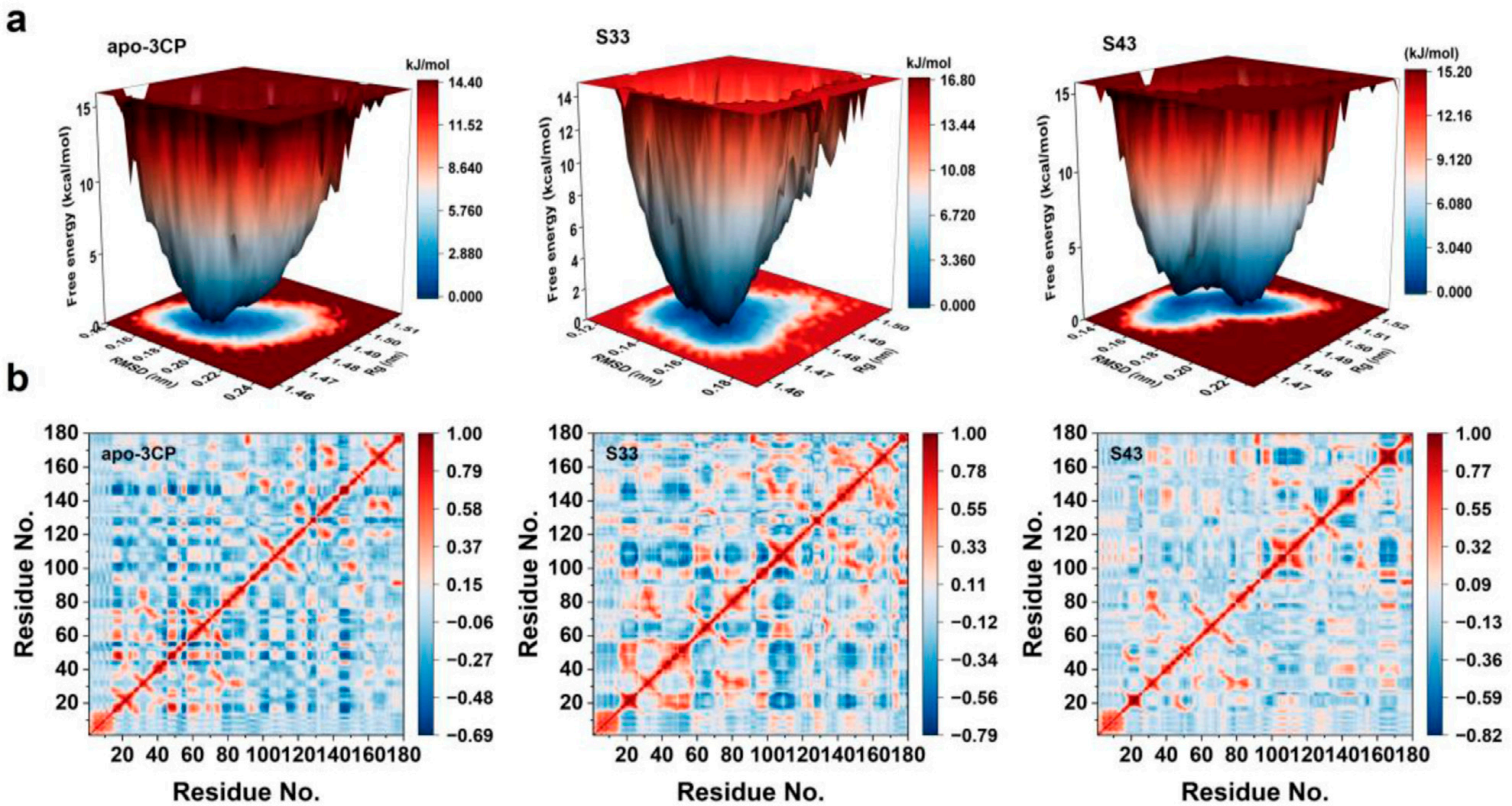
FEL and DCCM analyses of apo-3Cpro, **S33**, and **S43** systems. **(a)** Free energy landscapes for apo-3Cpro, **S33** and **S43** systems, projected onto RMSD and radius of gyration (Rg) to visualize conformational stability and metastable states. **(b)** DCCM of the Cα atoms of apo-3Cpro, **S33** and **S43** systems, showing correlated (red) and anti-correlated (blue) motions between residue pairs during the simulation.

The free energy landscapes (FEL) for the apo-3Cpro, **S33**, and **S43** systems were constructed using RMSD and Rg as reaction coordinates to characterize the conformational stability and dynamics of the protein systems (Figure 8a). The FEL of the apo-3Cpro system displayed a broader and more shallow energy basin, suggesting the presence of multiple metastable states. This observation indicates greater conformational flexibility in the absence of ligand binding, allowing the protein to sample a wider range of conformational states. In contrast, the FEL for the **S33** and **S43** systems showed narrower and deeper energy minima, indicative of enhanced structural stability. The pronounced single energy well in both ligand-bound systems reflects a more restricted conformational landscape, which is likely a result of the stabilizing effects of ligand interactions. Among the two ligand-bound systems, **S33** exhibited a slightly deeper energy well compared to **S43**, suggesting that **S33** may induce a more stable binding conformation. Overall, the FEL analysis highlights the significant influence of ligand binding on the protein’s energetic and conformational properties. The transition from a flexible apo state to a more rigid and stabilized ligand-bound state underscores the importance of ligand interactions in modulating protein dynamics and stability (Menezes et al., 2025).

The dynamic cross-correlation matrices (DCCM) for the apo-3Cpro, **S33**, and **S43** systems (Figure 8b) were analyzed to assess the residue-residue correlation patterns across the simulation (Yao et al., 2008). The apo-3Cpro system exhibited a more dispersed and less organized correlation pattern, with relatively weaker correlations between residues, as indicated by the dominance of light blue regions. This suggests that the absence of ligand binding resulted in increased flexibility and reduced coordinated motion within the protein structure. In contrast, the **S33** and **S43** systems displayed enhanced residue-residue correlations, as evident from the increased intensity of red and blue regions. These results indicate that ligand binding promoted more coordinated motions across the protein. Notably, the correlation intensity was more pronounced in certain regions, suggesting that ligand binding stabilized specific residue interactions, likely contributing to the overall structural rigidity of the protein. Furthermore, the diagonal elements of the DCCM, which represent intra-residue correlations, remained consistent across all systems. However, off-diagonal elements, reflecting inter-residue interactions, were more prominent in the ligand-bound systems. This observation highlights the role of ligand binding in establishing long-range communication pathways within the protein structure. Overall, the comparative analysis of the DCCM underscored the stabilizing effect of ligand binding on the protein’s dynamic behavior, enhancing both local and global residue correlations.

#### 3.4.4 Protein-ligand interactions

**FIGURE 9:**
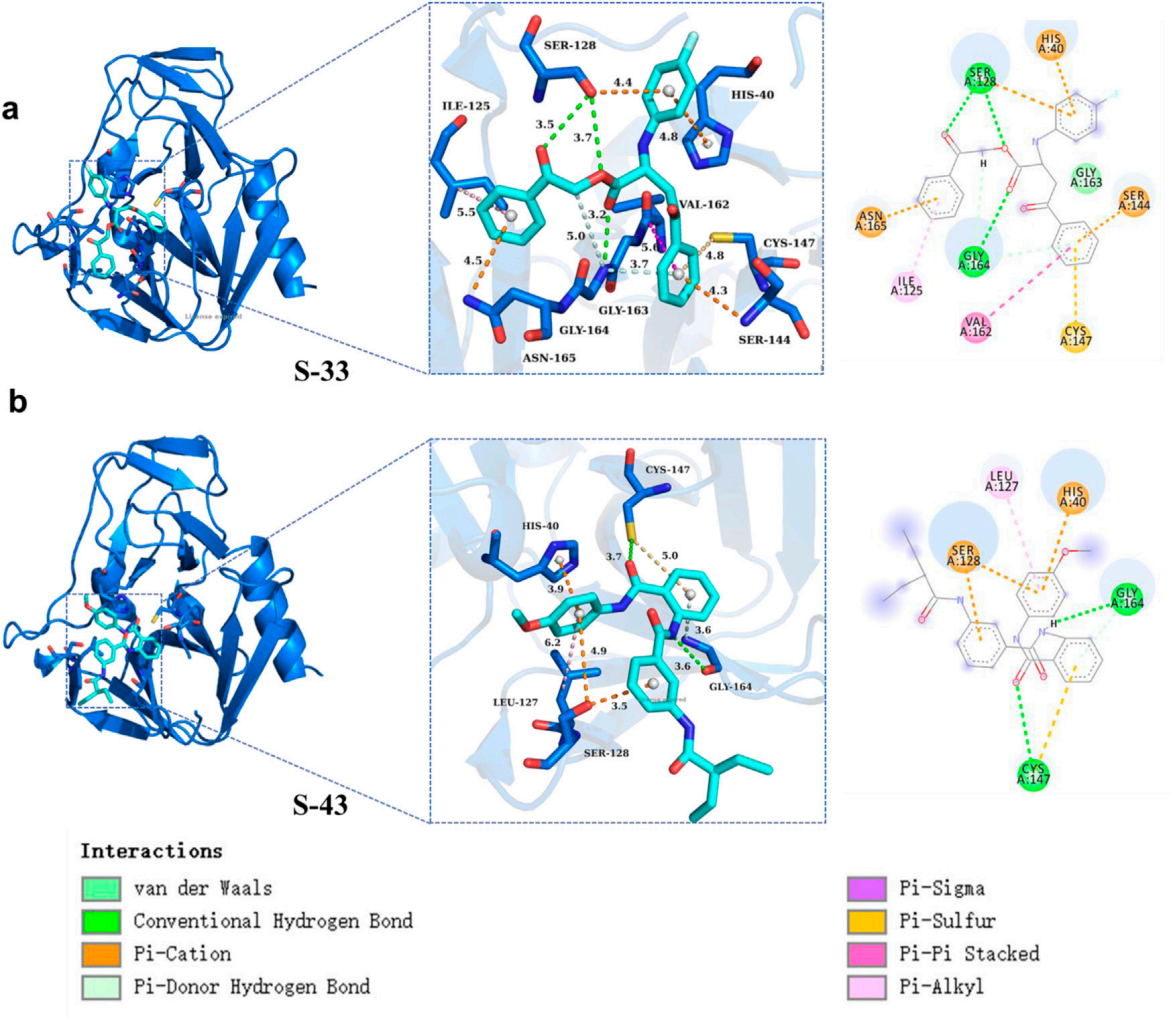
Protein ligand interactions in S33 and S43. **(a)** Three dimensional and two-dimensional interaction diagrams between S33 and ligand. **(b)** Three dimensional and two-dimensional interaction diagrams of S43 with ligands.

In this study, compound **S33** exhibited stable binding at the receptor binding site through multiple interaction modes with various amino acid residues (Figure 9a). It formed π-cation interactions with residues HIS40, SER128, SER144, and ASN165, which contributed to enhancing the stability of the compound within the receptor binding site, particularly in regions surrounding cationic amino acid residues (Pouria and Afshin, 2020). Additionally, compound **S33** established further interactions through conventional hydrogen bonds with residues SER128 and GLY164. The formation of these hydrogen bonds helped stabilize the positioning of the ligand in the receptor binding site, improving both binding specificity and strength. Compound **S33** also interacted with VAL162 via amide-π stacking interactions, further optimizing the binding mode and enhancing the stability of the ligand. This interaction was mediated by the π electron cloud of the aromatic ring and the amide group. Moreover, compound **S33** formed π-alkyl interactions with the alkyl side chain of residue ILE125, which facilitated the interaction between the aromatic ring and alkyl chain. This interaction reduced spatial clashes between the ligand and receptor, promoting better accommodation of the binding site. Finally, compound **S33** formed additional stabilization through a π-donor hydrogen bond with GLY164, where the π electron cloud of the aromatic ring interacted with the nitrogen atom of the hydrogen bond acceptor, further enhancing the ligand’s binding strength and stability.

Furthermore, compound **S43** formed a π-sulfur interaction between its aromatic ring and the sulfur atom of residue CYS147. This interaction maintained an optimal distance between the π electron cloud of the aromatic ring and the sulfur atom of CYS147, which contributed to the stability of the compound at the receptor binding site. Compound **S43** also established conventional hydrogen bonds with residues CYS147 and GLY164, further stabilizing the ligand-receptor interaction. In terms of hydrophobic interactions, the aromatic ring of **S43** formed π-alkyl interactions with the alkyl side chain of residue LEU127, enhancing the stability of the ligand within the binding site, particularly in hydrophobic regions. Additionally, compound **S43** formed a π-donor hydrogen bond with the amino nitrogen of GLY164, which further strengthened the ligand-receptor interaction. Finally, compound **S43** formed π-cation interactions with residues HIS40 and SER128, further contributing to the stability of the ligand-receptor complex (Figure 9b). In summary, compounds **S33** and **S43** established complex and diverse interaction networks within the receptor binding site through multiple interaction types, including π-sulfur, conventional hydrogen bonds, amide-π stacking, π-alkyl, and π-donor hydrogen bonds. These interactions collectively enhanced the binding affinity and stability of the compounds, providing valuable structural insights for future drug design.

#### 3.4.5 Binding free energy analysis

**FIGURE 10:**
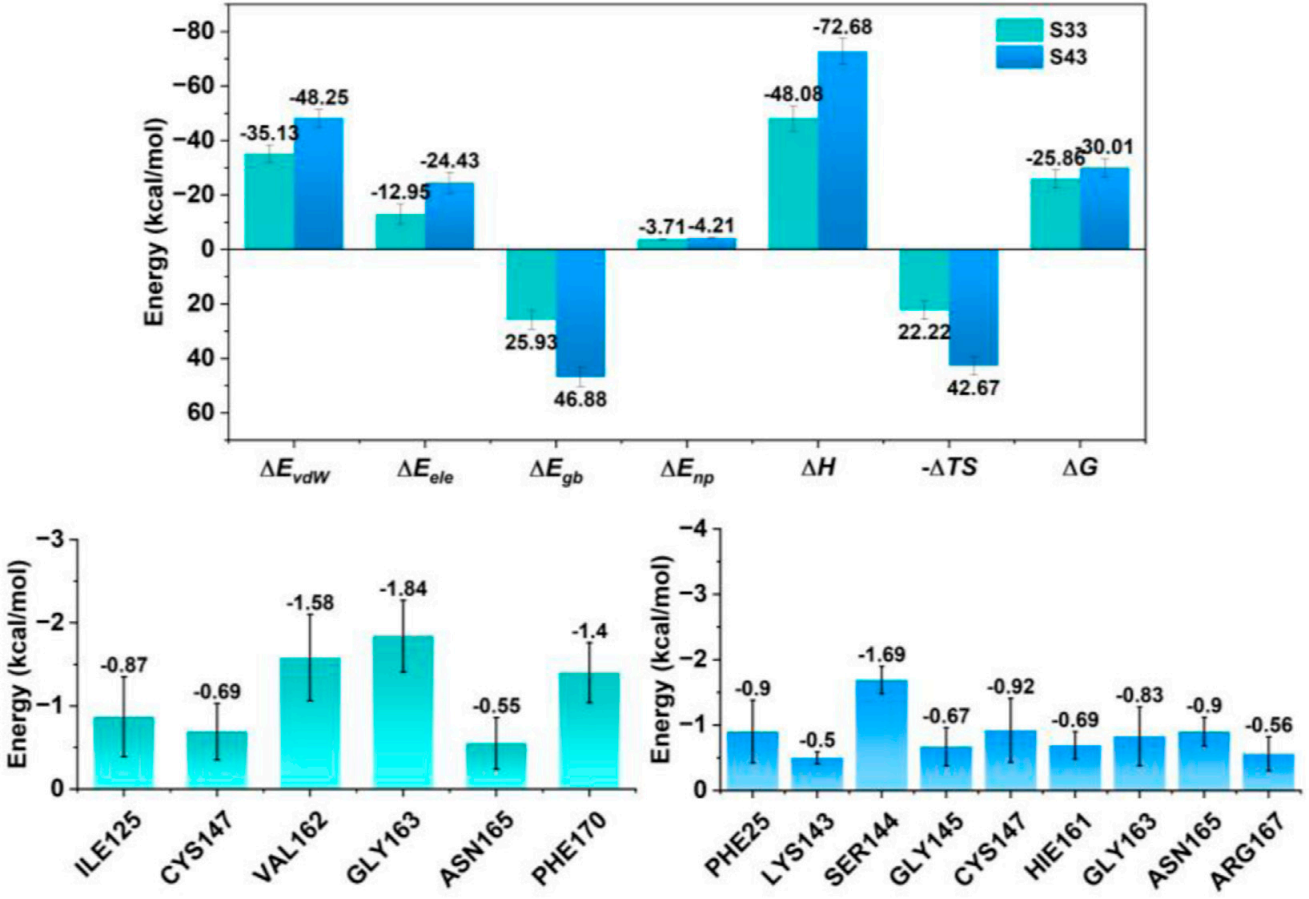
Energy contribution of residues near the binding site for each complex and total individual energy terms for each complex. The upper panel presents total energy components, including ΔE_vdW_, ΔE_ele_, ΔE_gb_, ΔE_np_, ΔH, (–TΔS), and ΔG. The lower panels display the residue-level energy contributions near the binding site for each compound.

In the present study, molecular dynamics simulations were employed to elucidate the interactions of compounds **S33** and **S43** with the HRV-14 3Cpro. A thorough examination of the energetic parameters was conducted, encompassing van der Waals forces (∆E_vdW_), electrostatic interactions (∆E_ele_), polar solvation (∆E_gb_), nonpolar solvation (∆E_np_), enthalpy change (∆H), entropy change (-T∆S), and free energy change (∆G). Both compounds **S33** and **S43** demonstrated significant negative values in van der Waals forces, with **S33** at −35.13 kcal/mol and **S43** at −48.25 kcal/mol, suggesting robust attractive interactions with the 3Cpro. The electrostatic contributions were also favorable, with **S33** and **S43** showing −12.95 kcal/mol and −24.43 kcal/mol, respectively, indicating that both compounds formed stable electrostatic interactions with the enzyme. In the case of polar solvation, while both compounds faced energy expenditures, **S43**’s value of 46.88 kcal/mol pointed to a potentially higher affinity for the protein, despite the apparent increase in energy due to solvation. Nonpolar solvation effects were minimal for both compounds, with **S33** at −3.71 kcal/mol and **S43** at −4.21 kcal/mol, suggesting that the hydrophobic interactions were well-maintained. The enthalpy change (∆H) for both compounds was negative, with **S33** at −48.08 kcal/mol and **S43** at −72.68 kcal/mol, indicating that the binding processes were exothermic and thus thermodynamically favorable. The entropy change (-T∆S) for **S33** and **S43** was 22.22 kcal/mol and 42.67 kcal/mol, respectively, which could imply a structured binding environment that might enhance the stability of the protein-ligand complex. The free energy change (∆G) for both compounds was negative, with **S33** at −25.86 kcal/mol and **S43** at −30.01 kcal/mol, underscoring the spontaneity and stability of the binding interactions. The residue-level analysis revealed that GLY163 and SER144 made substantial negative energy contributions to the binding of both **S33** and **S43**, highlighting their roles in stabilizing the protein-ligand interactions (Figure 10). In conclusion, the molecular dynamics simulations provided evidence that both **S33** and **S43** are potent inhibitors of 3Cpro, with favorable energetics that contribute to their binding stability. The insights gained from this study are instrumental for the rational design of novel antiviral agents targeting 3Cpro. The findings presented herein provide valuable structural and energetic insights for the future development of antiviral therapies targeting HRV-14 3Cpro.

### 3.5 DFT analysis

**FIGURE 11:**
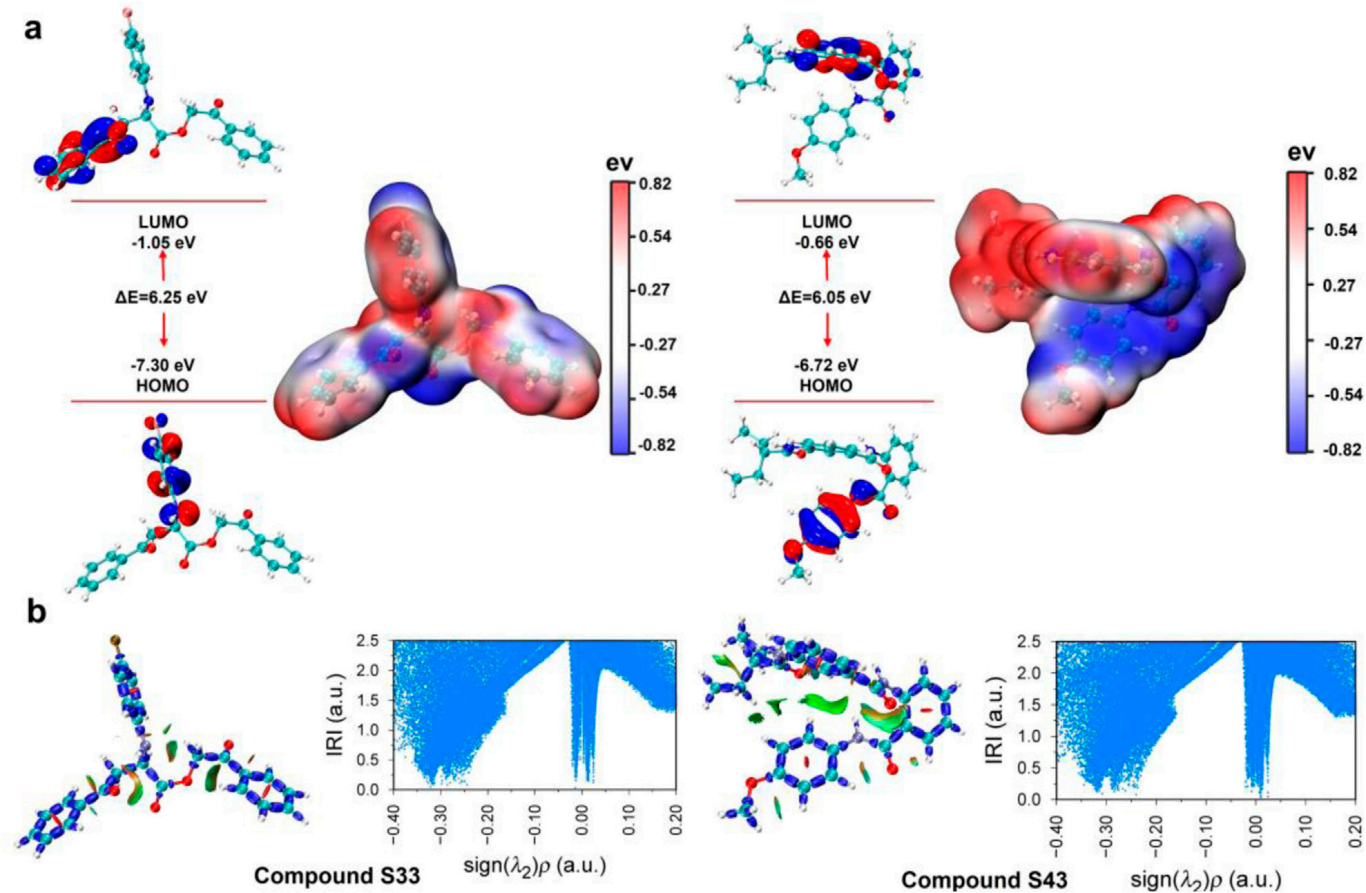
Frontier molecular orbital and IRI of compounds **S33** and **S43**. **(a)** HOMO-LUMO energy plot and electron density surface map of compounds **S33** and **S43**. **(b)** IRI analyses and IRI vs. sign (λ_2_) ρ scatter plot of c compounds **S33** and **S43** at the M06-2X/def2-TZVP level.

Understanding the electronic properties and molecular interactions of potential candidates is critical for evaluating their stability and reactivity. To this end, the HOMO-LUMO energy levels, electron density surface maps, and interaction region indicator (IRI) analyses of compounds **S33** and **S43** were investigated at the M06-2X/def2-TZVP level (Figure 11). Compound **S33** exhibited a HOMO energy of −7.30 eV and a LUMO energy of −1.05 eV, resulting in a HOMO-LUMO gap (ΔE) of 6.25 eV. In contrast, compound **S43** displayed a slightly lower ΔE of 6.05 eV, with a HOMO energy of −6.72 eV and a LUMO energy of −0.66 eV. The smaller HOMO-LUMO gap of **S43** suggests slightly enhanced electronic reactivity compared to **S33** (Figure 11a). Our results show that **S43**, which has the lowest HOMO–LUMO gap and the most favorable binding free energy (−30.01 kcal/mol), also exhibits the strongest inhibitory activity (IC_50_ = 2.33 ± 0.50 μM). In contrast, **S33** shows a slightly higher HOMO–LUMO gap and a slightly less favorable binding free energy (−25.86 kcal/mol). This parallel trend supports the idea that electronic properties derived from DFT can complement docking-based binding energy predictions, providing an additional layer of validation for the ligand’s binding potential.

The electron density surface maps further revealed distinct charge distributions: **S33** showed localized electron density around the conjugated core, while **S43** exhibited a more delocalized electron density, which may contribute to its reactivity and interaction capabilities. To evaluate non-covalent interactions, IRI analyses and IRI versus sign (λ_2_)ρ scatter plots were performed (Figure 11b). Both compounds displayed significant regions of attractive interactions, as indicated by high IRI values and negative sign (λ_2_)ρ regions. These findings highlight the presence of stabilizing interactions, particularly in **S43**, where broader and more pronounced interaction regions were observed. Overall, both **S33** and **S43** demonstrated favourable electronic properties and stable interaction profiles. While **S43** showed slightly higher electronic reactivity due to its smaller HOMO-LUMO gap and more delocalized electron distribution, the results suggest that both compounds can be considered promising candidates for further development and optimization.

## 4 Conclusion

In this study, we successfully identified and characterized novel non-covalent inhibitors of HRV-14 3Cpro through a comprehensive approach that included molecular docking, *in vitro* assays, MD simulations, and DFT analyses. These findings provide a significant scientific basis and practical insights for developing antiviral therapies targeting HRV-14. Through by layer screening of XP GScore, MM/GBSA, and Strain Energy modules using molecular docking technology, we identified 44 potential compounds from the Topscience and TargetMol (United States) database of one million compounds. ADMET analysis indicated that the majority of these compounds exhibited favorable pharmacokinetic and toxicological profiles. Further *in vitro* activity assays demonstrated that compounds **S21**, **S33**, **S34**, and **S43** exhibited good inhibitory activity, with **S33** and **S43** showing particularly significant effects, obtaining IC_50_ values of 11.32 ± 0.71 μM and 2.33 ± 0.5 μM, respectively, suggesting they are promising candidates for further development. Subsequent MD simulations revealed that both **S33** and **S43** enhanced the structural stability of HRV-14 3Cpro while reducing its flexibility and internal dynamics. PCA analysis highlighted significant conformational changes induced by ligand binding, indicating that **S33** and **S43** promoted a more stable and compact structure of HRV-14 3Cpro, which is conducive to enhanced inhibitory action.

FEL and DCCM analyses corroborated the results of the MD simulations, confirming that binding of **S33** and **S43** led to a transition of the protein structure to a more rigid and less flexible conformation. DFT calculations aligned with the MD simulations, demonstrating that **S33** and **S43** effectively inhibited 3Cpro by stabilizing its structure and hindering enzymatic activity. Further DFT analysis revealed favorable electronic properties, with HOMO-LUMO energy gaps (∆E) of 6.25 eV for **S33** and 6.05 eV for **S43**, indicating that **S43** has a higher reactivity. The electron density map and IRI analysis emphasized strong stabilizing interactions, with **S43** exhibiting a broader interaction range, suggesting greater potential for antiviral development. Additionally, the protein-ligand interaction studies highlighted robust and diverse interactions formed between **S33** and **S43** and critical active site residues of 3Cpro, including hydrogen bonds, van der Waals forces, π-π stacking, and π-sulfur interactions. These interactions significantly enhanced the binding affinity and stability of the compounds within the active site. The results from MD simulations and DFT analyses offer important guidance for the future design of 3Cpro inhibitors. The MD simulations revealed that compounds **S33** and **S43** not only stabilize the active site but also reduce overall protein flexibility an effect particularly valuable for improving target engagement and minimizing resistance from conformational shifts. Additionally, residue-level energy decomposition identified key amino acids such as SER144, GLY164, and HIS40 as central to ligand binding, suggesting these residues should be prioritized in future pharmacophore modeling or structure-based optimization. From a quantum chemical perspective, the HOMO-LUMO gap and IRI analysis of **S43** highlighted enhanced electronic reactivity and broader non-covalent interaction potential, indicating that increasing electron delocalization may further improve binding efficiency. Collectively, these insights suggest that optimizing interactions at specific hot-spot residues and fine-tuning electronic properties could yield more potent and selective non-covalent inhibitors.

Despite the promising outcomes, this study has several limitations. Firstly, the current evaluation of antiviral activity was limited to enzymatic assays, and no cell-based infection model was used to validate the ability of the compounds to inhibit HRV-14 replication in a biological context. Secondly, ADMET and toxicity data were obtained through computational predictions, which, while informative, require experimental confirmation. Thirdly, all structural analyses were performed using the HRV-14-14 3Cpro crystal structure, which may not capture the full diversity of HRV-14 serotypes. These limitations highlight the need for further validation and broadened experimental testing to fully establish the clinical potential of the identified compounds.

In summary, this study represents a significant advancement in the identification and characterization of non-covalent inhibitors for 3Cpro, particularly compounds **S33** and **S43**, both of which show immense potential for further development as antiviral agents. Looking ahead, future efforts will focus on the structural optimization of lead compounds **S33** and **S43** to further enhance their potency, selectivity, and pharmacokinetic properties. Specific modifications around key interacting moieties such as π-stacking and hydrogen bonding groups will be explored to improve binding affinity and metabolic stability. Additionally, cell-based antiviral assays will be conducted to evaluate the compounds’ efficacy in blocking HRV-14 replication. Promising candidates will then proceed to pharmacokinetic profiling and preclinical toxicity studies to assess their therapeutic potential *in vivo*. These next steps aim to translate the current *in silico* and *in vitro* findings into viable antiviral drug candidates.

## Data Availability

The original contributions presented in the study are included in the article/Supplementary Material, further inquiries can be directed to the corresponding authors.
