# Supplementary material for: Discovery of non-covalent rhinovirus 3Cpro inhibitors by molecular docking, in vitro assays, molecular dynamics simulations and DFT analyses
Source: Front Pharmacol. 2025 May 16;16:1560571. doi: 10.3389/fphar.2025.1560571 (PMC12122428; doi:10.3389/fphar.2025.1560571)
Supplement: Supplementary file 1 [file DataSheet1.docx]

# *Supplementary Material*

**Discovery of non-covalent rhinovirus 3C protease inhibitors by molecular docking, *in vitro* assays, molecular dynamics simulations and DFT analyses**

Susu Zhang ^a b†^, Keli Zong ^b †^, Jiajun Ruan ^a^, Xiaojing Liu ^b^, Xu Zhao ^c^, Youzhi Zhang ^b^ *, Chun Hu ^a^ *, and Xingzhou Li ^b^ *

^a^ Key Laboratory of Structure-based Drug Design & Discovery (Ministry of Education), Shenyang

Pharmaceutical University,103 Wenhua Road, Shenyang 110016, China

*^b^Beijing Institute of Pharmacology and Toxicology, 27 Taiping Road, Beijing 100850, China*

*^c^Department of Hepatology, Fifth Medical Center of Chinese PLA General Hospital, 100 West Fourth Ring Road, Beijing100071, China*

^†^These authors contribute equally; * Correspondence author.

* Correspondence author e-mail: bcczyz@163.com, [chunhu@syphu.edu.cn](mailto:chunhu@syphu.edu.cn), and lixz@bmi.ac.cn


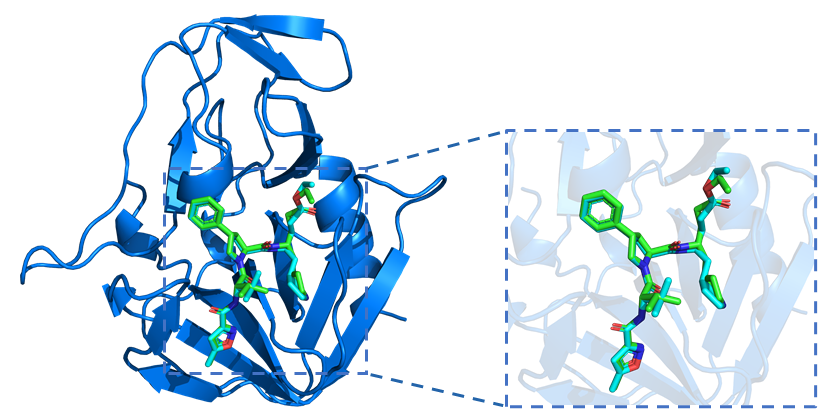


**Fig. S1** Alignment of redocked (blue) and crystallographic (green) ligand in 3Cpro active site validating docking precision.

**Table S1** The XP Gscore and MMGBSA energies of 44 top compounds and **compound 14**

| Compound | Structure | XP GScore (kcal/mol) | MM/GBSA  *∆G_bind_* (kcal/mol) | Strain Energy  (kcal/mol) |
| --- | --- | --- | --- | --- |
| **S1** |  | $-$6.756 | $-$48.64 | 3.68 |
| **S2** |  | $-$6.766 | $-$41.36 | 8.98 |
| **S3** |  | $-$6.035 | $-$44.30 | 3.12 |
| **S4** |  | $-$5.667 | $-$26.48 | 2.22 |
| **S5** |  | $-$5.730 | $-$29.13 | 3.9 |
| **S6** |  | $-$5.815 | $-$36.97 | 8.53 |
| **S7** |  | $-$7.451 | $-$40.95 | 2.48 |
| **S8** |  | $-$7.874 | $-$52.1 | 0.91 |
| **S9** |  | $-$5.657 | $-$27.33 | 2.43 |
| **S10** |  | $-$6.862 | $-$33.01 | 0.58 |
| **S11** |  | $-$5.474 | $-$35.18 | 5.49 |
| **S12** |  | $-$8.359 | $-$48.26 | 3.75 |
| **S13** |  | $-$5.895 | $-$33.6 | 7.56 |
| **S14** |  | $-$7.693 | $-$37.61 | 10.98 |
| **S15** |  | $-$5.121 | $-$47.64 | 1.26 |
| **S16** |  | $-$6.128 | $-$27.92 | 5.29 |
| **S17** |  | $-$7.050 | $-$57.55 | 2.04 |
| **S18** |  | $-$5.604 | $-$38.76 | 9.53 |
| **S19** |  | $-$5.634 | $-$31.99 | 2.99 |
| **S20** |  | $-$6.596 | $-$38.21 | 6.69 |
| **S21** |  | $-$6.640 | $-$45.70 | 4.04 |
| **S22** |  | $-$6.102 | $-$49.23 | 2.89 |
| **S23** |  | $-$7.387 | $-$54.98 | 8.95 |
| **S24** |  | $-$5.656 | $-$17.37 | 4.46 |
| **S25** |  | $-$5.521 | $-$32.27 | 9.66 |
| **S26** |  | $-$6.670 | $-$45.39 | 12.17 |
| **S27** |  | $-$5.910 | $-$50.88 | 5.58 |
| **S28** |  | $-$6.402 | $-$60.79 | 6.59 |
| **S29** |  | $-$6.728 | $-$39.30 | 1.21 |
| **S30** |  | $-$7.657 | $-$47.67 | 8.69 |
| **S31** |  | $-$5.243 | $-$33.59 | 5.44 |
| **S32** |  | $-$6.693 | $-$49.15 | 7.99 |
| **S33** |  | $-$6.268 | $-$47.67 | 2.33 |
| **S34** |  | $-$6.653 | $-$44.50 | 6.05 |
| **S35** |  | $-$5.147 | $-$29.56 | 4.45 |
| **S36** |  | $-$5.955 | $-$36.7 | 10.4 |
| **S37** |  | $-$5.662 | $-$36.98 | 1.15 |
| **S38** |  | $-$6.200 | $-$29.34 | 13.01 |
| **S39** |  | $-$7.399 | $-$47.10 | 7.72 |
| **S40** |  | $-$7.910 | $-$36.14 | 3.88 |
| **S41** |  | $-$5.885 | $-$36.48 | 6.54 |
| **S42** |  | $-$8.039 | $-$45.49 | 8.37 |
| **S43** |  | $-$5.261 | $-$41.84 | 3.66 |
| **S44** |  | $-$5.680 | $-$27.63 | 3.16 |
| **14** |  | $-5.226$ | $-$29.39 | 0.04 |

**Table S2** The ADMET properties of the 44 top compounds

| Compound | ABS | Water solubility | BBB | P-gp | LD50 (mg/kg) | Toxicity Class |
| --- | --- | --- | --- | --- | --- | --- |
| **S1** | High | Moderately | Yes | Yes | 1000 | Ⅳ |
| **S2** | High | Moderately | No | Yes | 540 | Ⅳ |
| **S3** | High | Soluble | No | Yes | 800 | Ⅳ |
| **S4** | High | Moderately | No | No | 1460 | Ⅳ |
| **S5** | High | Moderately | No | Yes | 1240 | Ⅳ |
| **S6** | High | Moderately | No | Yes | 200 | Ⅳ |
| **S7** | High | Moderately | No | Yes | 1000 | Ⅲ |
| **S8** | High | Moderately | No | Yes | 300 | Ⅲ |
| **S9** | High | Moderately | No | Yes | 1460 | Ⅳ |
| **S10** | High | Moderately | Yes | No | 1600 | Ⅳ |
| **S11** | High | Moderately | No | Yes | 1240 | Ⅳ |
| **S12** | Low | Moderately | No | Yes | 1000 | Ⅳ |
| **S13** | High | Soluble | No | Yes | 1000 | Ⅳ |
| **S14** | High | Moderately | No | Yes | 572 | Ⅳ |
| **S15** | High | Moderately | Yes | No | 1000 | Ⅳ |
| **S16** | High | Moderately | No | Yes | 1646 | Ⅳ |
| **S17** | High | Moderately | No | No | 4000 | V |
| **S18** | High | Moderately | No | Yes | 1000 | Ⅳ |
| **S19** | High | Soluble | No | Yes | 707 | Ⅳ |
| **S20** | High | Moderately | No | Yes | 1460 | Ⅳ |
| **S21** | High | Moderately | Yes | No | 1800 | Ⅳ |
| **S22** | High | Moderately | Yes | No | 250 | Ⅲ |
| **S23** | Low | Moderately | No | No | 1000 | Ⅳ |
| **S24** | High | Moderately | No | Yes | 1460 | Ⅳ |
| **S25** | High | Moderately | No | No | 560 | Ⅳ |
| **S26** | High | Moderately | No | No | 1100 | Ⅳ |
| **S27** | High | Soluble | Yes | Yes | 1500 | Ⅳ |
| **S28** | High | Moderately | No | Yes | 1000 | Ⅳ |
| **S29** | High | Soluble | No | Yes | 650 | Ⅳ |
| **S30** | High | Soluble | No | Yes | 1000 | Ⅳ |
| **S31** | High | Moderately | Yes | No | 2000 | Ⅳ |
| **S32** | High | Moderately | No | Yes | 1500 | Ⅳ |
| **S33** | High | Moderately | Yes | Yes | 3000 | V |
| **S34** | High | Moderately | Yes | No | 1750 | Ⅳ |
| **S35** | High | Moderately | Yes | No | 3000 | V |
| **S36** | High | Moderately | No | No | 4000 | V |
| **S37** | High | Moderately | Yes | No | 500 | Ⅳ |
| **S38** | High | Soluble | No | Yes | 2300 | V |
| **S39** | High | Moderately | Yes | No | 1500 | Ⅳ |
| **S40** | High | Soluble | No | Yes | 1000 | Ⅳ |
| **S41** | Low | Moderately | No | Yes | 1000 | Ⅳ |
| **S42** | High | Moderately | Yes | No | 1500 | Ⅳ |
| **S43** | High | Moderately | No | No | 1000 | Ⅳ |
| **S44** | High | Moderately | No | No | 1000 | Ⅳ |

**Table S3** The toxicity properties of the 44 top compounds.

| Compound | Hepatotoxicity | Neurotoxicity | Respiratory toxicity | Carcinogenicity | Immunotoxicity | Mutagenicity |
| --- | --- | --- | --- | --- | --- | --- |
| **S1** | Inactive (0.90) | active (0.64) | active (0.88) | Inactive (0.53) | active  (0.71) | Inactive (0.70) |
| **S2** | Inactive (0.76) | active (0.60) | active (0.66) | Inactive (0.70) | Inactive (0.61) | Inactive (0.61) |
| **S3** | Inactive (0.84) | active (0.51) | active (0.77) | Inactive (0.56) | active  (0.87) | Inactive (0.64) |
| **S4** | Inactive (0.51) | active (0.91) | active (0.79) | active  (0.57) | Inactive (0.89) | Inactive (0.59) |
| **S5** | Inactive (0.66) | active (0.75) | active (0.85) | Inactive (0.58) | Inactive (0.90) | Inactive (0.58) |
| **S6** | Inactive (0.72) | active (0.66) | active (0.72) | Inactive (0.59) | Inactive (0.96) | Inactive (0.56) |
| **S7** | active (0.53) | active (0.61) | active (0.60) | Inactive (0.62) | active  (0.85) | Inactive (0.60) |
| **S8** | Inactive (0.54) | active (0.82) | active (0.80) | Inactive (0.83) | Inactive (0.94) | Inactive (0.62) |
| **S9** | Inactive (0.64) | active (0.88) | active (0.75) | active  (0.68) | Inactive (0.99) | Inactive (0.58) |
| **S10** | active (0.60) | active (0.76) | active (0.70) | active  (0.54) | active  (0.97) | active (0.58) |
| **S11** | Inactive (0.65) | active (0.80) | active (0.89) | Inactive (0.61) | Inactive (0.91) | Inactive (0.62) |
| **S12** | Inactive (0.50) | active (0.50) | active (0.60) | Inactive (0.62) | Inactive (0.87) | Inactive (0.61) |
| **S13** | Inactive (0.87) | Inactive (0.57) | active (0.66) | Inactive (0.64) | Inactive (0.95) | Inactive (0.86) |
| **S14** | Inactive (0.79) | active (0.66) | active (0.76) | Inactive (0.63) | Inactive (0.55) | Inactive (0.68) |
| **S15** | active (0.51) | active (0.65) | active (0.76) | active  (0.58) | Inactive (0.58) | active (0.74) |
| **S16** | Inactive (0.83) | active (0.83) | active (0.82) | Inactive (0.72) | Inactive (0.86) | Inactive (0.67) |
| **S17** | Inactive (0.83) | Inactive (0.58) | active (0.65) | Inactive (0.62) | Inactive (0.99) | Inactive (0.74) |
| **S18** | Inactive (0.74) | active (0.60) | active (0.66) | Inactive (0.59) | active  (0.94) | Inactive (0.61) |
| **S19** | Inactive (0.87) | active (0.50) | active (0.67) | Inactive (0.65) | Inactive (0.95) | Inactive (0.59) |
| **S20** | Inactive (0.52) | active (0.84) | active (0.81) | active  (0.62) | Inactive (0.97) | Inactive (0.61) |
| **S21** | Inactive (0.82) | Inactive (0.66) | active (0.57) | Inactive (0.56) | Inactive (0.87) | Inactive (0.76) |
| **S22** | Inactive (0.71) | active (0.57) | active (0.78) | Inactive (0.67) | active  (0.99) | Inactive (0.69) |
| **S23** | active (0.52) | active (0.65) | active (0.63) | active  (0.59) | Inactive (0.99) | Inactive (0.73) |
| **S24** | Inactive (0.56) | active (0.90) | active (0.75) | active  (0.73) | Inactive (0.99) | Inactive (0.62) |
| **S25** | Inactive (0.60) | active (0.88) | active (0.86) | Inactive (0.56) | Inactive (0.97) | Inactive (0.51) |
| **S26** | Inactive (0.81) | Inactive (0.54) | active (0.55) | Inactive (0.57) | Inactive (0.96) | Inactive (0.75) |
| **S27** | Inactive (0.78) | active (0.68) | active (0.66) | Inactive (0.62) | Inactive (0.99) | Inactive (0.69) |
| **S28** | Inactive (0.87) | active (0.52) | active (0.83) | Inactive (0.60) | active  (0.56) | Inactive (0.70) |
| **S29** | Inactive (0.60) | active (0.80) | active (0.87) | Inactive (0.52) | active  (0.98) | Inactive (0.52) |
| **S30** | active (0.57) | active (0.55) | active (0.51) | active  (0.58) | Inactive (0.99) | Inactive (0.56) |
| **S31** | Inactive (0.76) | active (0.51) | active (0.52) | Inactive (0.62) | Inactive (0.88) | Inactive (0.73) |
| **S32** | Inactive (0.79) | active (0.67) | active (0.66) | Inactive (0.63) | Inactive (0.99) | Inactive (0.70) |
| **S33** | Inactive (0.66) | Inactive (0.55) | Inactive (0.50) | Inactive (0.59) | Inactive (0.99) | Inactive (0.68) |
| **S34** | Inactive (0.79) | active (0.50) | active (0.54) | Inactive (0.62) | Inactive (0.91) | Inactive (0.74) |
| **S35** | Inactive (0.68) | active (0.55) | active (0.70) | Inactive (0.52) | Inactive (0.87) | Inactive (0.66) |
| **S36** | Inactive (0.51) | Inactive (0.81) | Inactive (0.85) | Inactive (0.62) | Inactive (0.62) | Active (0.86) |
| **S37** | active (0.53) | active (0.71) | active (0.76) | active  (0.55) | Inactive (0.91) | active (0.74) |
| **S38** | Inactive (0.89) | active (0.52) | active (0.68) | Inactive (0.65) | Inactive (0.92) | Inactive (0.71) |
| **S39** | Inactive (0.78) | active (0.62) | Inactive (0.53) | Inactive (0.67) | Inactive (0.99) | Inactive (0.79) |
| **S40** | Inactive (0.78) | active (0.54) | active (0.72) | Inactive (0.59) | active  (0.92) | Inactive (0.66) |
| **S41** | Inactive (0.55) | Inactive (0.66) | active (0.53) | Inactive (0.64) | active  (0.99) | Inactive (0.61) |
| **S42** | Inactive (0.77) | active (0.53) | Inactive (0.50) | Inactive (0.66) | Inactive (0.98) | Inactive (0.79) |
| **S43** | Inactive (0.56) | active (0.56) | active (0.56) | active  (0.54) | Inactive (0.52) | Inactive (0.63) |
| **S44** | Inactive (0.57) | active (0.70) | active (0.80) | Inactive (0.59) | Inactive (0.99) | Inactive (0.59) |

**Table S4** Inhibition of HRV 3CP Activity by 44 top compounds

| compound | Inhibition %  HRV 3Cpro  @ 50 $\mu$M | compound | Inhibition %  HRV 3Cpro  @ 50 $\mu$M |
| --- | --- | --- | --- |
| **S1** | -46.7 | **S23** | -12.0 |
| **S2** | -34.3 | **S24** | -66.5 |
| **S3** | -22.4 | **S25** | -70.7 |
| **S4** | -13.3 | **S26** | -60.0 |
| **S5** | 29.6 | **S27** | -68.9 |
| **S6** | -36.8 | **S28** | -72.4 |
| **S7** | 72.7 | **S29** | -59.5 |
| **S8** | -34.4 | **S30** | -68.1 |
| **S9** | 0.50 | **S31** | -56.0 |
| **S10** | -67.1 | **S32** | -51.7 |
| **S11** | 25.7 | **S33** | 96.5 |
| **S12** | 31.3 | **S34** | 75.5 |
| **S13** | -48.1 | **S35** | -37.4 |
| **S14** | 13.2 | **S36** | 38.3 |
| **S15** | -128.9 | **S37** | -272.3 |
| **S16** | -79.56 | **S38** | -29.9 |
| **S17** | 4.25 | **S39** | 12.6 |
| **S18** | -25.8 | **S40** | -44.4 |
| **S19** | -72.6 | **S41** | -82.0 |
| **S20** | -64.7 | **S42** | -70.4 |
| **S21** | 80.5 | **S43** | 88.7 |
| **S22** | -36.6 | **S44** | -7.3 |

a: % inhibition = Kinase activity inhibited, determined at 50 μM of compound concentration.
